# DNA damage induction during localized chronic exposure to an insoluble radioactive microparticle

**DOI:** 10.1038/s41598-019-46874-6

**Published:** 2019-07-17

**Authors:** Yusuke Matsuya, Yukihiko Satou, Nobuyuki Hamada, Hiroyuki Date, Masayori Ishikawa, Tatsuhiko Sato

**Affiliations:** 1 Nuclear Science and Engineering Center, Research Group for Radiation Transport Analysis, Japan Atomic Energy Agency (JAEA), 2-4 Shirakata, Tokai, Ibaraki 319-1195 Japan; 2 Collaborative Laboratories for Advanced Decommissioning Science (CLADS), Japan Atomic Energy Agency (JAEA), 790-1 Otsuka, Motooka, Tomioka, Fukushima 979-1151 Japan; 3 Radiation Safety Research Center, Nuclear Technology Research Laboratory, Central Research Institute of Electric Power Industry (CRIEPI), 2-11-1 Iwado-kita, Komae, Tokyo 201-8511 Japan; 4 Faculty of Health Sciences, Hokkaido University, Kita-12 Nishi-8, Kita-ku, Sapporo, Hokkaido 060-0812 Japan

**Keywords:** Cell biology, Computational science

## Abstract

Insoluble radioactive microparticles emitted by the incident at the Fukushima nuclear power plant have drawn keen interests from the viewpoint of radiation protection. Cs-bearing particles have been assumed to adhere in the long term to trachea after aspirated into respiratory system, leading to heterogeneous dose distribution within healthy tissue around the particles. However, the biological effects posed by an insoluble radioactive particle remain unclear. Here, we show cumulative DNA damage in normal human lung cells proximal and distal to the particle (β-ray and γ-ray-dominant areas, respectively) under localized chronic exposure in comparison with uniform exposure. We put a Cs-bearing particle into a microcapillary tip and placed it onto a glass-base dish containing fibroblast or epithelial cells cultured *in vitro*. A Monte Carlo simulation with PHITS code provides the radial distribution of absorbed dose-rate around the particle, and subsequently we observed a significant change in nuclear γ-H2AX foci after 24 h or 48 h exposure to the particle. The nuclear foci in the cells distal to the particle increased even under low-dose-rate exposure compared with uniform exposure to ^137^Cs γ-rays, which was suppressed by a treatment with a scavenger of reactive oxygen species. In contrast, such focus formation was less manifested in the exposed cells proximal to the particle compared with uniform exposure. These data suggest that the localized exposure to a Cs-bearing particle leads to not only disadvantage to distal cells but also advantage to proximal cells. This study is the first to provide quantitative evaluation for the spatial distribution of DNA double strand breaks after the heterogeneous chronic exposure to a Cs-bearing particle in comparison with uniform Cs exposure.

## Introduction

Large amounts of artificial radionuclides were released after the incident at the Fukushima Dai-ichi Nuclear Power Station (F1NPS) in 2011^1,2^, which have drawn keen interest of public in terms of radiation safety^3^. Insoluble microparticles containing radioactive cesium (Cs-bearing particles)^4^ have widely dispersed 20 km northwest and 170 km south of the F1NPS^5^. The radioactive particles were firstly isolated from soil near the F1NPS in 2016, the physical details of which have been accumulated^5,6^. There are two types of Cs-bearing particles: Type A in a high specific radioactivity and 1–10 μm diameter, and Type B in a low specific radioactivity and 70–400 μm diameter^6^. The particle type can be linked to source reactors, such that the Type A particle arose from the unit 2 or 3, Type B from the unit 1, both at F1NPS^6^. Concerning the particle feature, Cs-bearing particles have been assumed to adhere in a long term to trachea after aspirated into respiratory system due to its insolubility in the body fluid.

The risk of internal exposure by radioactive particles is one of great public concerns from the viewpoint of radiation protection^7,8^. The organ dose given by an intake of insoluble Cs-bearing particles is expected to differ from that of soluble particles even with the same radioactivity on account of different biokinetics in the human body. So, Manabe, *et al*. have proposed a new biokinetic model considering the insolubility feature of Cs-bearing particles in addition to conventional biokinetics^9^. Furthermore, microenvironment of cells would heavily rely on the energy deposition which is concentrated in the region close to Cs-bearing particles^5^. Although it has been confirmed that micron scale is not necessarily needed for estimating the biological effects by soluble ^134^Cs and ^137^Cs^10^, those by insoluble Cs-bearing particle have not yet been evaluated. Thus, the estimation of the biological effects only from organ dose would be insufficient, so it is necessary to investigate the biological effects under localized energy deposition with the *in vitro* experiments.

Among various types of ionizing radiation damage to DNA, evaluation of DNA double-strand breaks (DSBs) is of importance^11^, which can be detected by the use of histone H2AX phosphorylated on serine 139 (γ-H2AX foci) that surround DSBs^12–15^. The *in vitro* experiments under heterogeneous exposure have shown that radiation effects appear not only in irradiated cells (via targeted effects)^16^ but also in non-irradiated cells due to intercellular communication from irradiated cells (via non-targeted effects)^17–21^. Non-targeted effects have been observed for various endpoints such as DNA damage induction^22,23^ and cell survival^24,25^. For localized chronic energy deposition by a Cs-bearing particle, the DNA damage arising from intercellular communication is suspected to accumulate^26,27^. In contrast, there are some reports on radioresistance resulting from the benefit of intercellular communication^28–30^. However, there is no report of the effects on cells in the vicinity of a Cs-bearing particle after the long-term exposure. Therefore, we here set out to focus on DSBs induction under the local exposure to Cs-bearing particles.

This study aims to quantify the number of DSBs induced after exposure to a Cs-bearing particle. Assuming adherence of the particles to lung tissue in the long term, we used two normal human lung cell lines (WI-38 fibroblast cells and HBEC-3KT bronchial epithelial cells), and detected the DSBs by means of γ-H2AX focus formation assay. In this study, we evaluated the DNA damage responses under heterogeneous exposure by a Cs-bearing particle, compared with conventional uniform Cs exposure.

## Materials and Methods

### Cell culture

Primary normal human diploid lung fibroblast (WI-38) cell line and human bronchial epithelial cell line immortalized with hTERT and CDK4 (HBEC-3KT) were used, assuming that a Cs-bearing particle adheres to lung tissue. WI-38 cells (CCL-75) and HBEC-3KT cells (CRL-4051) were obtained from American Type Culture Collection (ATCC, Manassas, VA, USA). WI-38 cells were maintained in Dulbecco’s modified Eagle’s medium/Nutrient Mixture F-12 (DMEM/F12) (D8437, Sigma Life Science) supplemented with 10% fetal bovine serum (FBS, Equitech-Bio Inc.), as previously described^31^. HBEC-3KT cells were maintained in bronchial epithelial cell medium (3211NZ, ScienCell). These cells were maintained at 37°C in a humidified atmosphere of 5% CO_2_ in air. The cells were seeded onto *ϕ*12-mm or *ϕ*27-mm glass-based dishes (3911-035 or 3960-035, IWAKI) for detecting DSBs after exposure. Confluent monolayer was used for experiments.

### γ-H2AX focus formation assay

The induced DSBs were quantitatively detected by immunostaining with an antibody against γ-H2AX. After exposure, the cells were fixed in 4% paraformaldehyde for 10 min on ice. The cells rinsed with phosphate buffered saline (PBS) were permeabilized in 0.2% Triton X-100 in PBS for 5 min, and blocked in 1% bovine serum albumin (BSA) in PBS for 30 min. The cells were incubated at 4°C overnight with a primary antibody against γ-H2AX (ab26350, Abcam) diluted 1:400 by a 1% BSA in PBS, and rinsed with a 1% BSA in PBS three times. Subsequently, the cells were incubated for 2 h in the dark at room temperature with Alexa Fluor 594-conjugated goat-anti-mouse IgG H&L (ab150116, Abcam) diluted 1:250 by a solution of 1% BSA in PBS, as a secondary antibody. After rinsed with a 1% BSA in PBS three times, the cells were incubated with 1 μg/ml DAPI solution (62248, Thermo Fisher Scientific) for 15 min. After rinsed with methanol, γ-H2AX foci were detected by using a High Standard all-in-one fluorescent microscope (model BZ-9000; Keyence, Osaka, Japan). The number of nuclear foci (≥80 cells) was counted by using the Image J software^32,33^, as reported previously^15^.

### Control method for a Cs-bearing particle and exposure condition

As a sample of Cs-bearing particles to observe changes in the level of DNA damage, we used a single Type B particle composed of 92.4% ^137^Cs with 469.2 Bq and 7.6% ^134^Cs with 38.5 Bq, which was collected within the area around F1NPS (the sample ID assigned as CF-01)^6^. This particle is rare one with relative high radioactivity among Cs-bearing particles. In this study, the Type B particle was put inside the microcapillary with a 20 μm tip inner diameter (MP-020, Micro Support Co., Ltd) to locate the particle in a cell culture dish. It should be noted that the tip was closed to prevent the back flow of cell culture medium. During the design phase of experiments, we tested several candidate materials for enclosing the particle. In consideration of less toxicity to cells during 24 h incubation with material (Supplementary I, Figs S1 and S2) and an easy operability at micron scale, we decided to employ the microcapillary.

**Figure 1:**
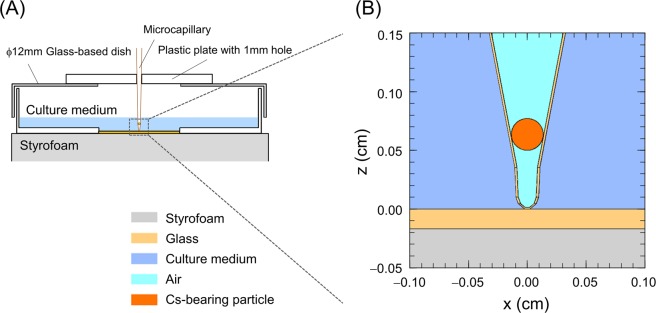
Geometry for positioning of a Cs-bearing particle. (**A**) How to place the microcapillary containing the Type B particle with 505.7 Bq, (**B**) Microenvironment described by the PHITS code. The tip of the capillary was closed, the inside of which was filled with air. The diameter and position of the Cs-bearing microparticle were measured by using the Image J software.

To fix the capillary on the center of a *ϕ*12-mm glass-based dish as schematized in Fig. 1A, we prepared the dish lid with a 1 mm diameter hole, with which the capillary can easily be inserted or removed. The lid was firmly fixed to the culture dish for a period from the start of the exposure to the end of detecting DSBs by using fluorescence microscope. However, when performing the γ-H2AX focus formation assay, we have to exchange the capillary containing the Type B particle to one without radioactive particle. We therefore evaluated the positional deviation of the capillary tip when exchanging the capillary. The standard deviation of the tip position on the surface of the dish was 69.6 μm from the cold-run trials performed ten times.

For the DNA damage experiment with the Cs-bearing particle, we used the microcapillary containing the Type B particle and the modified glass-based dish as shown in Fig. 1A. The exposure time was set as 24 h and 48 h. To evaluate the impact of intercellular signalling, experiments for 24 h exposure case were also performed in the presence of the free radical scavenger, dimethyl sulphoxide (DMSO, Fujifilm Wako, Japan) at a final concentration of 1% added 2 h prior to the beginning of exposure.

### Calculation of dose-rate deposited by a Cs-bearing particle

To obtain the relationship between absorbed dose-rate around the Cs-bearing particle and the number of nuclear foci, we calculated the radial distribution of absorbed dose-rate from the tip of the capillary. We calculated the absorbed dose-rate by using a Monte Carlo simulation code, Particle and Heavy Ion Transport Code System (PHITS ver. 3.08)^34^ considering RI source database (ICRP07)^35^. The mode of electron gamma shower (EGS)^36^ was adapted in the PHITS simulation, whilst the cut off energies for photons and electrons were set as 1.0 keV.

The geometry for simulation was consistent with that used in the actual experiment, as illustrated in Fig. 1B. In Fig. 1B, the Type B particle with 505.7 Bq containing ^137^Cs and ^134^Cs is assumed as a simple sphere, which is located at the position of (*x*, *y*, *z*) = (0, 0, 635.5) in μm. The properties of the Type B particle considered in the PHITS calculation are listed in Table 1. As shown in Fig. 1B, the inside of the capillary was filled with air because the tip was closed so that culture medium could not enter. The diameter and position of the Type B particle were given by the measurement from the picture of enclosed capillary tip with the Image J software analysis^32,33^. The particle diameter was calculated by averaging the major axis and the minor axis. The composition of capillary was assumed as SiO_2_ with density of 2.2 g/cm^3^.

**Table 1:** Properties of a Type B particle incorporated into the PHITS.

Radioisotopes (activity ratio)	Activity (Bq)	Diameter (μm)	Composition	Density (g/cm^3^)
^134^Cs (7.6%)	38.5	271.0	SiO_2_	2.2
^137^Cs (92.4%)	469.2			

The activities for ^134^Cs and ^137^Cs were deduced as of March 11, 2011. The shape of the Type B particle is assumed as sphere for simplicity and the diameter is given by the measurement with the Image J software analysis. The composition of the particle was assumed to be SiO_2_ in 2.2 g/cm^3^.

### Irradiation setup for uniform exposure and half-field exposure

We performed the experiments under uniform exposure to ^137^Cs γ-rays to compare the number of nuclear foci under localized exposure to the Cs-bearing particle with that under uniform exposure. The cells were exposed to γ-rays emitted from a ^137^Cs source for 24 h. The dose-rate in air was measured, and we converted it to that in water according to The International Atomic Energy Agency (IAEA) Technical Report Series No. 277^37^. The dose-rate was changed according to inverse square law of distance to be 0.001, 0.005, 0.010, 0.051, 0.096, 0.444, 1.005 and 7.286 Gy/day. We also checked these various dose-rates in culture medium by the calculation with PHITS.

To further discuss the biological effects of heterogeneous exposure, the experiment after a half-field exposure was conducted. In this experiment, *ϕ*27-mm glass-based dishes containing the cells were placed at the dose boundary between in-field and out-of-field (5 cm away from isocenter) so as to expose 50% cells to 1.0 Gy of 6 MV-linac X-rays. The field size and the depth from the surface were set as 10 × 10 cm^2^ and 10 cm, respectively. The dose-rate in water at 10 cm depth (isocenter) was measured according to Japanese Standard Dosimetry 12^38^. After the irradiation, we measured nuclear foci of the in-field cells placed at 1 cm inside from the dose boundary (4 cm away from isocenter in the in-field region). The nuclear foci detected under half-field exposure were compared with those under uniform field with 6 MV-linac X-rays.

### Statistics

Significant differences of nuclear foci in number were evaluated by using a multiple comparison method, the Tukey-Kramer test. Based on the statistical test, we evaluated the number of DSBs among the localized exposure with Cs-bearing particle, half-field exposure and uniform exposure under culture conditions in the presence or absence of 1% DMSO.

## Results and Discussions

### Radial distribution of dose-rate around a Type B particle

**Figure 2:**
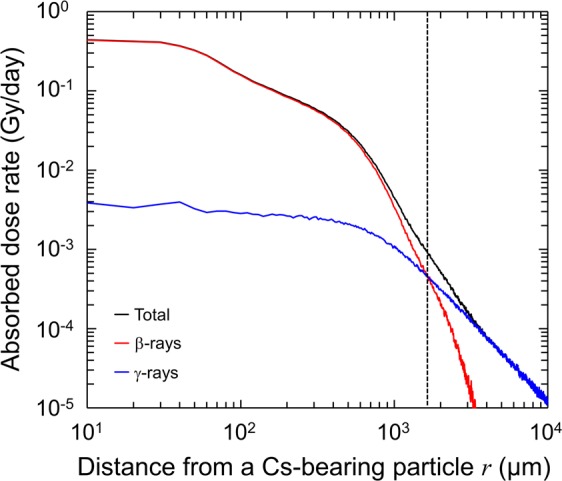
Absorbed dose-rates as a function of distance from a Type B particle. The absorbed dose-rate was calculated by using a Monte Carlo code with PHITS, in which the dose in the area within 1.65 mm (presented as a dotted line) away from the particle is deposited dominantly by β-rays in comparison with dose by γ-rays.

The dose-rate absorbed during the chronic exposure to the Type B particle was calculated by using the Monte Carlo code of PHITS, due to difficulty in dose measurement at micron level. Figure 2 shows the relationship between the radial distance away from the particle and the absorbed dose-rate in Gy/day, where red, blue and black solid lines represent the absorbed doses originated from β-rays, γ-rays and sum of β-rays and γ-rays, respectively. As shown in Fig. 2, β-rays mainly contribute to the energy deposition compared with γ-rays in the region within 1.65 mm from the particle. On the other hand, secondary electron produced by γ-rays dominantly deposits their energy in the area 1.65 mm away from the particle, leading to a less absorbed dose-rate below 0.902 mGy/day as the total absorbed dose-rate by β-rays and γ-rays.

For the calculation, we set the density of composition SiO_2_ as 2.2 g/cm^3^. The increasing tendency of radioactivity as a function of the volume, however, has been reported to differ between Types A and B particles^6^, suggesting that the composition density of Type B particle is lower than that of Type A particle (lower than 2.2 g/cm^3^). Taking this into account, we also calculated absorbed dose-rate at various densities. However, the calculated maximum difference in absorbed doses directly under the particle at the density of 1.8, 1.4 and 1.0 g/cm^3^ was 9.1%, 17.4% and 28.3%, respectively, compared with the 2.2 g/cm^3^ case. This suggests that the influence of the different density of the Type B particle on biological effects is small enough (Supplementary II, Fig. S3).

### DSBs induced by exposure to a Cs-bearing particle

**Figure 3:**
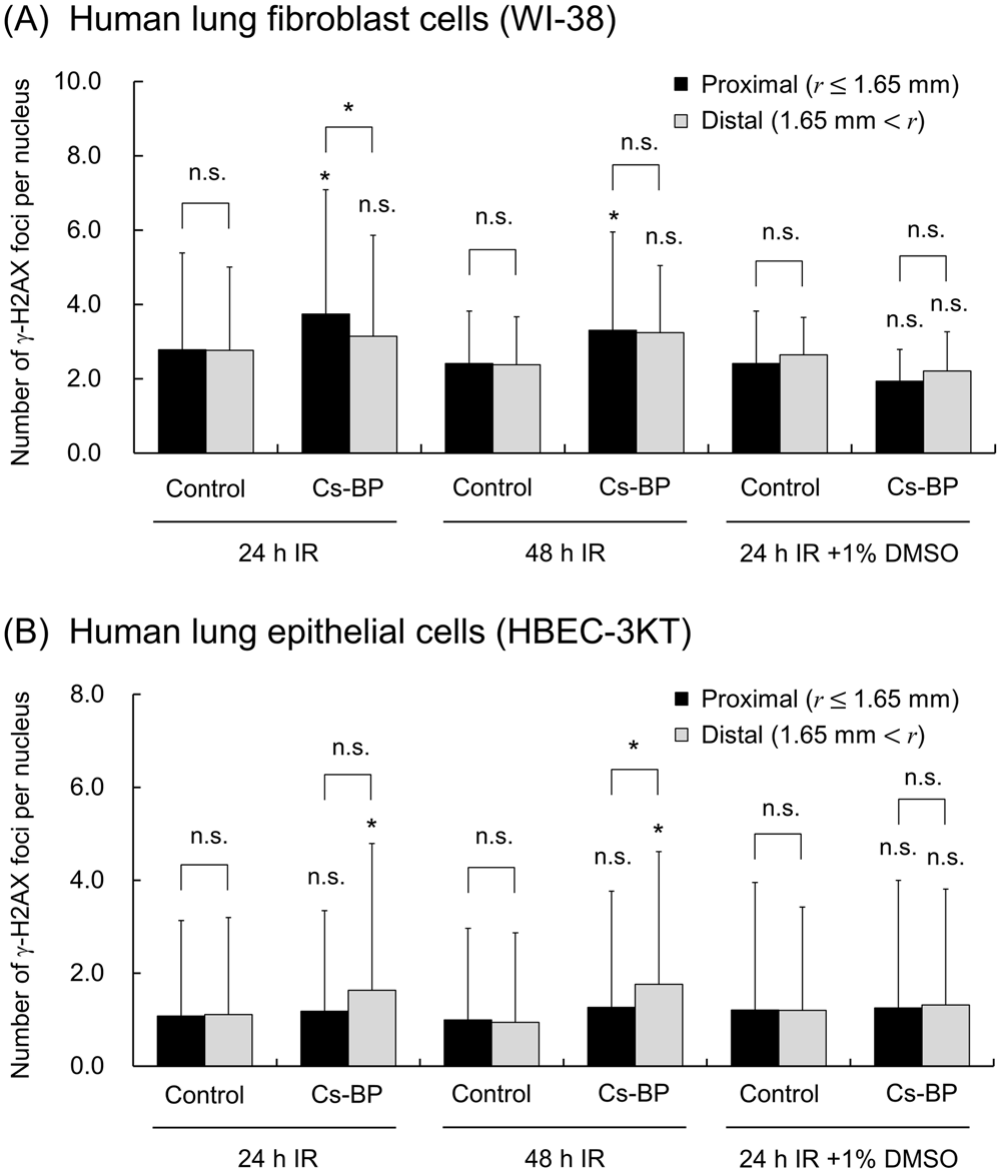
Cumulative γ−H2AX foci detected after Cs-bearing particle (Cs-BP) exposure. (**A**) Normal human lung fibroblast cells (WI-38). (**B**) Normal human lung epithelial cells (HBEC-3KT). The error bars indicate the standard deviations. The exposure time was set as 24 h or 48 h, and the irradiation is noted as IR in this Fig. 3. To check the impact of intercellular communication, 1% DMSO was added 2 h prior to the beginning of the 24 h exposure. The symbol of * and n.s. represents 5% significant difference and no significant difference, respectively, vs the corresponding control (non-irradiated) group.

Figure 3 shows the results of γ-H2AX focus formation assay, where black and grey bars are the nuclear foci in the cells located in the area within 1.65 mm (β-ray-dominant area) and those in the area away from 1.65 mm (γ-ray-dominant area), respectively. The symbol of * represents the 5% significant differences between the two groups. As shown in Fig. 3, a significant increase in the focus number after 24 h or 48 h exposure in comparison with non-irradiated (control) cells was observed only in the proximal region of WI-38 cells (β-ray dominant area closer than 1.65 mm) and in the distal region of HBEC-3KT cells (γ-ray dominant area farther than 1.65 mm). The low-dose-rate exposure by the Cs-bearing particle implies that the detected foci are attributable to non-targeted signalling from exposed cells close to the particle. To test whether reactive oxygen species (ROS) through intercellular communication contributes to this effect or not, we examined the effect of 1% DMSO. As shown in the right most two columns in Fig. 3. We found that 1% DMSO diminishes the DSB induction, suggesting that ROS as intercellular signals are involved in the enhancement of DSB induction under localized exposure to the Cs-bearing particle.

The enhanced DSB induction was also observed in the cells away from the Cs-bearing particle, suggesting that the intercellular signals can travel up to 3 mm away from the particle. Concerning this signal diffusion distance, the previous study on non-targeted effects *in vitro* shows that the γ-H2AX foci can be detected at early stage (30 min) in the non-irradiated cells 10 mm away from irradiated cells^39^. Our experimental result is consistent with this *in vitro* experimental result on non-targeted effects^39^. However, the three dimensional tissue model shows the limited signal-traveling range, i.e., 90–100 μm for calcium wave^40^, and ~800 μm for apoptosis signals^41^. To precisely grasp the impact range for signalling effects in the human body, further investigation is warranted.

Among the intercellular signals^42–44^, we focused on ROS as the final messenger of the signals^20,21^, to check whether or not the intercellular signals are dominant factors for enhanced induction of DSBs. DMSO was used for scavenging the signal; however, it can also cancel indirect effects such as ∙OH (chemical reaction)^45^ in irradiated cells. There are lots types of the signals, calcium, nitric oxide (NO), ROS, interleukin 6 (IL-6), etc^46–50^. Of these, IL-6 production has been reported both in several cultured normal human lung cell lines *in vitro*^51,52^ and in mice and rats *in vivo*^53,54^, supportive of the signalling events occurring in normal human lung cells following exposure to Cs-bearing particles. In order to further search major contributors, experiments with indene^39^ or aminoguanidine^23^ will be useful.

### Comparison between localized exposure and uniform exposure

**Figure 4:**
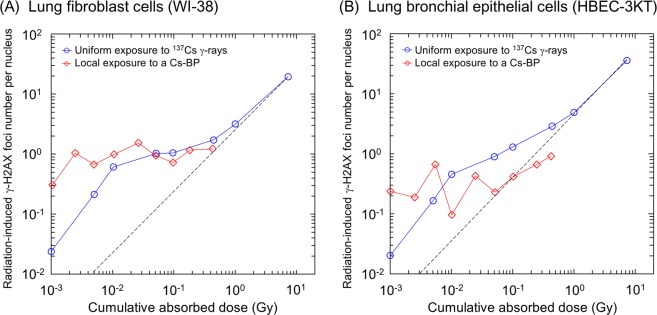
Comparison of DSB induction between localized exposure to Cs-bearing particle (Cs-BP) and uniform exposure. (**A**) Lung fibroblast cells (WI-38), (**B**) Lung bronchial epithelial cells (HBEC-3KT). The mean number of γ-H2AX foci detected after 24 h exposure to the Cs-BP was linked to absorbed dose calculated by PHITS. The solid lines are for eye guide. The dotted line represents the interpolation between high dose data with 7.29 Gy and the origin, corresponding to the prediction based on DNA-targeted effects. The mean number of foci per nucleus by the Cs-BP is intersecting with that by uniform exposure to ^137^Cs γ-rays in a dose range of about 50 mGy for WI-38 cells, about 5–10 mGy for HBEC-3KT cells.

We next evaluated the dependence of DSB induction on cumulative absorbed dose posed by local exposure to a Cs-bearing particle compared with the uniform exposure to ^137^Cs γ-rays. As shown Fig. 4, an increase in DSB induction by signalling effects could be checked in the low-dose range. Intriguingly, the average number of foci per nucleus by the Cs-bearing particle was intersecting with that by uniform exposure to ^137^Cs γ-rays in a dose range of about 50 mGy for WI-38, about 5–10 mGy for HBEC-3KT. This comparison shows reduced radiosensitivity in the cells exposed at higher dose-rate, suggesting a feedback by intercellular communication from non-irradiated cells leading to advantageous effects by virtue of intercellular signals^28–30^. In Fig. 4, the mean number of DSBs as a function of the cumulative dose was plotted owing to large uncertainties of nuclear γ-H2AX foci which may result from variation of nuclear dose and the cell-cycle dependent DNA amount per nucleus (as radiation target)^15,55^. Due to the uncertainty, there is also a possibility that the dose-response curve in the WI-38 cells exposed to the Cs-bearing particle coincides with that under the uniform exposure in high-dose range above about 50 mGy.

The mean number of DSBs in WI-38 cells after 24 h uniform exposure with 7.29 Gy by ^137^Cs γ-rays was 19.5 ± 13.1 per nucleus, whilst that in HBEC-3KT cells was 35.9 ± 20.4 per nucleus. The DSB induction surely competes with DNA repair function^56^ (non-homologous end joining, NHEJ^57^) during 24 h exposure. The comparison of the number of DSBs between WI-38 cells and HBEC-3KT cell suggests that repair rate of DSBs in HBEC-3KT cells is lower than that in WI-38 cells. To confirm this, we also examined DNA repair kinetics of DSBs after acute exposure to 1.0 Gy. It was certified the half-time for repair of DSBs for HBEC-3KT cells (3.29 h) is longer than that for WI-38 cells (2.11 h) (Supplementary III, Fig. S4).

Focusing on the low-dose range in Fig. 4, the unexpected number of DSBs was observed under uniform exposure. This tendency might be predominantly attributed to low-dose hyper-radiosensitivity (HRS) believed to result from failure to G_2_ arrest (cell-cycle problem)^58,59^. The precise mechanisms of low-dose HRS remain unclear, but the enhanced DSB under uniform exposure may be attributable to stacked HRS. For this, further investigation is necessary for clarifying low-dose-rate HRS problem.

Considering the DNA repair during exposure^56,57^, it is interesting to note that the nuclear foci under heterogeneous exposure by the Cs-bearing particle is smaller than that under the uniform exposure to ^137^Cs γ-rays. The reduced number of nuclear DSBs seems to be prominent in HBEC-3KT cells with longer repair-half time than WI-38 cells. In this regard, it is reasonable to suppose that the feedback of intercellular signals from non-exposed cells to exposed cells close to the Cs-bearing particle reduce the yield of DSBs. As for this hypothesis, we made the half-field experiments additionally to evaluate the initial number of DSBs after acute non-uniform exposure in both WI-38 and HBEC-3KT cells.

### Reduced yield of initial damage under heterogeneous exposure

**Figure 5:**
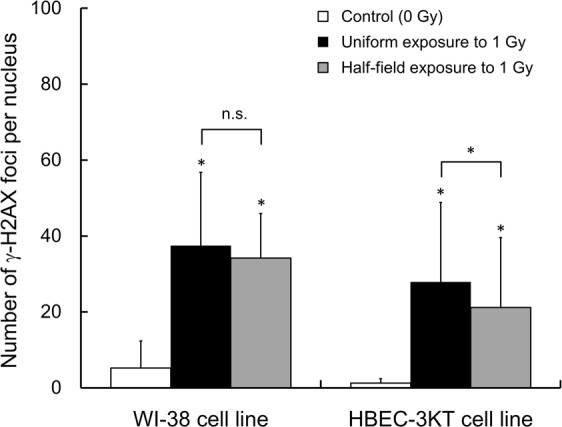
Reduced number of γ-H2AX foci under heterogeneous exposure. To demonstrate the non-uniform exposure, the half-field (with 50% in the culture dish exposed) experiment is conducted. The γ-H2AX foci were observed 30 min after irradiation with 6 MV-linac X-rays. The symbol of *and n.s. represents 5% significant difference and no significant difference, respectively.

Figure 5 shows the results by half-field experiments with constant 1.0 Gy, where the number of DSBs/cell/Gy was 32.2 ± 14.6 in WI-38 cells and 26.6 ± 14.8 in HBEC-3KT cells. Even though the detection of foci at 30 min detected after irradiation (early stage), the yield under the half-field exposure in both cells was reduced to 29.0 ± 9.70 per Gy (89.9 ± 50.6%) and 19.9 ± 13.0 per Gy (75.0 ± 64.4%), respectively. Such tendency implies that the initial yield of DSBs is modified independent of DNA repair function, suggesting “protective effects”^60^. It has been reported that the natural barrier from the stress (ROS) is induced under exposure at an environmental level^61^. The results obtained with the half-field experiments support that the reduced DNA damage induction is stacked in the cells exposed close to the Cs-bearing particle, resulting in the smaller DSB number compared with the uniform exposure.

## Conclusion

Given that a Cs-bearing radioactive particle chronically adheres to trachea after entry into the respiratory system, we investigated the spatial characteristics of cumulative DNA damage during the localized energy deposition by a Cs-bearing particle in comparison with uniform exposure. The dose at a cellular level was calculated by using the PHITS code, providing β-ray and γ-ray dominant regions as proximal and distal cells to a Cs-bearing particle, respectively. The experiments with two types of normal human lung cell lines (i.e., fibroblast and bronchial epithelial cells) exhibited an increase of DNA damage in the cells distal to a Cs-bearing particle (DNA damage enhancement by ROS) and a reduced yield of DNA damage induction in the cells proximal to a Cs-bearing particle (protective response). These results suggest that the local exposure to a Cs-bearing particle leads to increase of damage (disadvantage) to distal cells and reduction of damage (advantage) to proximal cells, suggesting the adequacy of risk estimation based on averaged organ dose. This study is the first evaluation of the relationship between absorbed dose-rate and cumulative DNA lesions under heterogeneous chronic exposure to a Cs-bearing particle in comparison to the uniform Cs exposure. Further studies are needed to understand the underlying mechanisms under non-uniform exposure as well as *in vivo* effects by such a particle.

## Supplementary information

Supplementary data
