# Supplementary material for: DNA damage induction during localized chronic exposure to an insoluble radioactive microparticle
Source: Sci Rep. 2019 Jul 17;9:10365. doi: 10.1038/s41598-019-46874-6 (PMC6637188; doi:10.1038/s41598-019-46874-6)
Supplement: Supplementary file 1 — Supplementary data [file 41598_2019_46874_MOESM1_ESM.pdf]

DNA damage induction during localized chronic exposure to an insoluble radioactive microparticle

Yusuke Matsuya[1*], Yukihiko Satou[2], Nobuyuki Hamada[3], Hiroyuki Date[4], Masayori Ishikawa[4], Tatsuhiko Sato[1]

[1]Nuclear Science and Engineering Center, Research Group for Radiation Transport Analysis, Japan Atomic Energy Agency (JAEA), 2-4 Shirakata, Tokai, Ibaraki, 319-1195, Japan
[2]Collaborative Laboratories for Advanced Decommissioning Science (CLADS), Japan Atomic Energy Agency (JAEA), 790-1 Motooka Ohtsuka, Tomioka Town, Futaba-gun, Fukushima, 979-1151, Japan
[3]Radiation Safety Research Center, Nuclear Technology Research Laboratory, Central Research Institute of Electric Power Industry (CRIEPI), 2-11-1 Iwado-kita, Komae, Tokyo, 201-8511, Japan
[4]Faculty of Health Sciences, Hokkaido University, Kita-12 Nishi-8, Kita-ku, Sapporo, Hokkaido 060-0812, Japan
* Corresponding author (matsuya.yusuke@jaea.go.jp)

Supplementary Information

I. *Selection of material for enclosing a Cs-bearing particle*

In the main body of the text, we employed a method to put a Type B radioactive particle containing Cs into a microcapillary (MP-020, Micro Support Co., Ltd) and placed the microcapillary onto the cell-containing glass base dish (3911–035, IWAKI). There are several material candidates to deliver the particle onto the dish, hand-made SiO_2 capillary (Fig. S1(I)), microcapillary (Micro Support Co., Ltd) (Fig. S1(II)), a tungsten needle (Fig. S1(III)), copper pin (Fig. S1(IV)), and carbon tape (Fig. S1(V)). At the design phase of experiments with a Type B particle, we checked cytotoxicity of the 24 h incubation with materials.

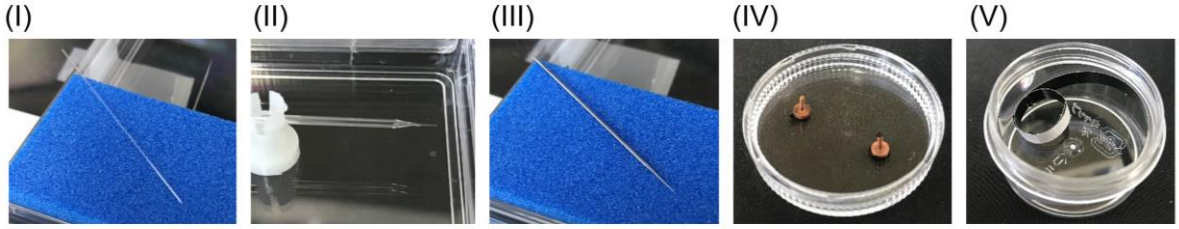

Figure S1 Picture of material candidates for delivering Cs-bearing particle onto culture dish: (I) for hand-made SiO_2 capillary, (II) for microcapillary (Micro Support Co., Ltd), (III) for a tungsten needle, (IV) for copper (Cu) pin, and (V) for carbon (C) tape.

We used primary normal human diploid lung fibroblast (WI-38) cells, and incubated the WI-38 cells with each material described above for 24 h. The positive control treated with 10 mM Mitomycin C (MMC) was also prepared as well as negative control (mock) group. The endpoint of cell toxicity was set to be DNA double-strand break (DSB) detection by means of γ-H2AX foci formation assay as reported previously.[1]

Figure S2 shows the results by the γ-H2AX focus formation assay, where the significance between the two groups was evaluated by using a multiple comparison method, the Tukey-Kramer test. As shown in Fig. S2, there was no significant DSB induction for (I) hand-made SiO_2 capillary, (II)

microcapillary and (III) a tungsten needle. Conventionally, Cs-bearing particles are handled by the combination of (IV) Cu pin and (V) C tape, however the combination induce the toxicity to cells during 24 h incubation, which means that the (IV) and (V) are not suitable for *in vitro* experiment of DSB detection.

As described in the main text, in consideration of the less toxicity to cells during 24 h incubation with materials (Fig. S2) and operability at micron scale, we selected the microcapillary with 20 μm tip inner diameter (MP-020, Micro Support Co., Ltd).

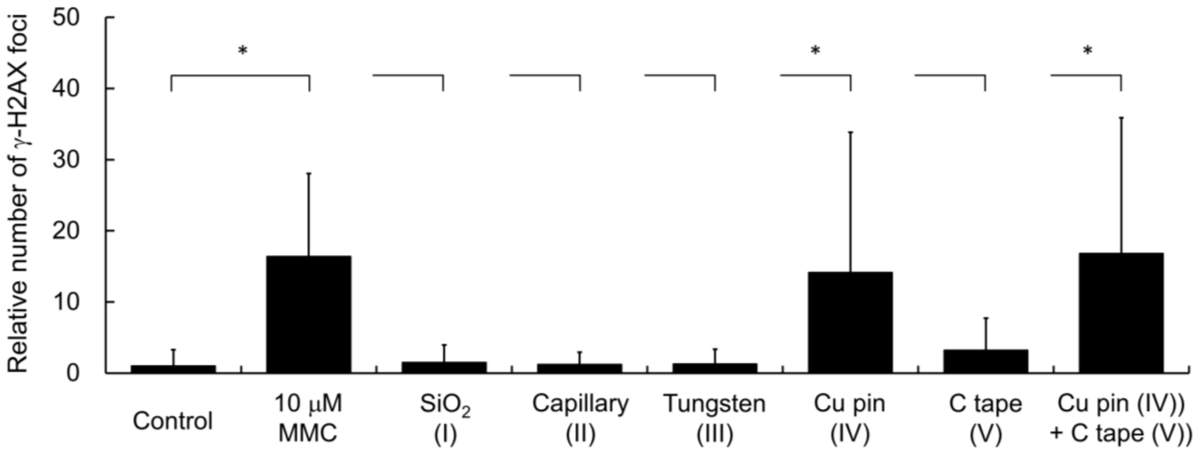

Figure S2 Cytotoxicity testing for selecting the materials to deliver a Cs-bearing particle onto the dish. The γ-H2AX foci formation assay was performed in WI-38 cells after 24 h incubation with each material. The roman numerals are linked to those in Fig. S1. We prepared a positive control treated with 10 mM Mitomycin C (MMC) as well as a negative control (mock) group. The significances were evaluated by using a multiple comparison method, the Tukey-Kramer test. The symbol * represents 5 % significant differences.

II. *Influence of composition density on absorbed dose-rate around a Type B particle*

Assuming that the density of SiO_2 in a Cs-bearing particle is 2.2 g/cm^3, we calculated the radial distribution of absorbed dose-rate around a Type B Cs-bearing particle with 505.7 Bq. However, there is a possibility that the density of Type B particle is lower than 2.2 g/cm^3, according to the previous report.[2] For this reason, we also calculated absorbed dose-rate for various densities of Type B particle.

To calculate the dose-rate around the particle, we used a Particle and Heavy Ion Transport Code System (PHITS ver. 3.08)[3] considering RI source database (ICRP07)[4] and the mode of electron gamma shower (EGS).[5] In the same manner as the calculation in the main text, the cut off energies for photons and electrons were set as 1.0 keV. The composition was set to be the same SiO_2 as the main text, but the we here changed the density to 2.2, 1.8, 1.4 and 1.0 g/cm^3.

Figure S3 shows the relationship between the density of Type B particle and relative absorbed dose-rate for the regions of 0-50 μm, 500-550 μm, 1000-1050 μm and 1500-1550 μm away from the particle. The calculated absorbed dose-rates were normalized by those at 2.2 g/cm^3. As shown in Fig. S3, the maximum difference of absorbed doses for the densities of 1.8, 1.4 and 1.0 g/cm^3 were 9.1%,

17.4% and 28.3%, respectively, compared with the 2.2 g/cm^3 case. As discussed in the main text, in comparison of nuclear foci between heterogenous exposure by the particle and uniform exposure by ^{137}Cs γ-rays, two types of DNA damage responses, i.e., the enhanced DSB induction in the cells distal to the particle and the reduced DSBs induction (protective effects) in the cells close to the particle were observed with the γ-H2AX focus formation assay. With the increment of absorbed dose-rate under lower density of the particle composition than 2.2 g/cm^3 (Fig. S3), whilst the signal-induced DSBs become less, the protective effects are more prominent than the result in main text (Fig. 4).

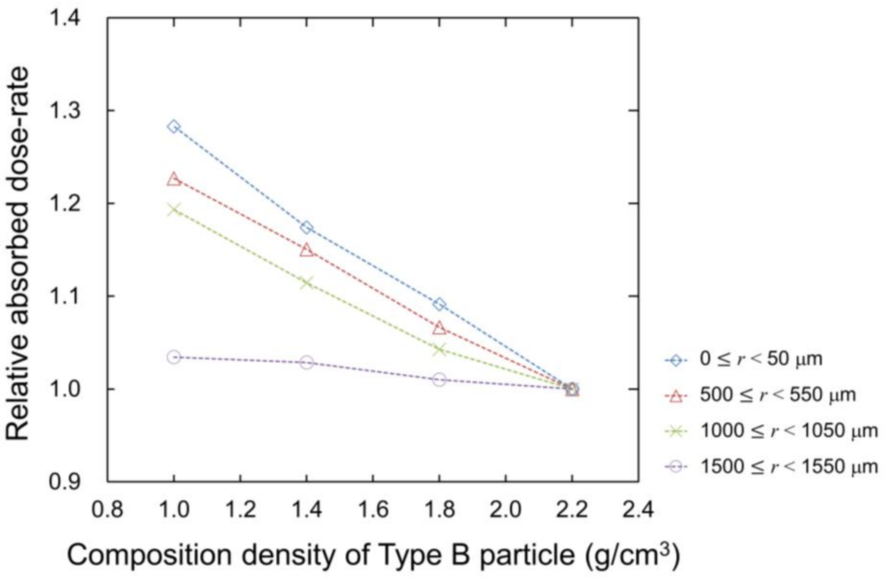

Figure S3 Absorbed dose-rate for various densities of Type B particle. Using a Particle and Heavy Ion Transport Code System (PHITS ver. 3.08)[3], we calculated the radial distribution of absorbed dose-rate for the density of 1.0, 1.4, 1.8 and 2.2 g/cm^3. The relative absorbed dose-rate for the regions of 0–50 μm, 500–550 μm, 1000–1050 μm and 1500–1550 μm away from the particle were shown. The calculated absorbed dose-rates were normalized by those for the case of 2.2 g/cm^3. The dotted lines are for eye guide.

III. *Half-time for DSB repair*

The number of DSBs induced after 24 h or 48 h exposure was quantitatively detected by means of γ-H2AX foci formation assay. According to the previous reports,[6-9] at least 20–30 DSBs are induced per cell per Gy. In contrast, the numbers of DSBs per nucleus after 1.0 Gy continuous exposure shown in the main text were 3.02 ± 5.25 in WI-38 cells and 4.89 ± 4.14 in human bronchial epithelial (HBEC-3KT) cells. This difference should be attributable to DNA repair during the exposure,[10,11] but the number of DSBs per HBEC-3KT cells was greater than that per WI-38 cells. So here, we added the experiments on DNA damage repair kinetics after acute exposure to X-rays.

WI-38 and HBEC-3KT cells were acutely exposed to 1.0 Gy with 6 MV-linac X-rays. The field size and the depth were set to be 10 × 10 cm^2 and 10 cm from the surface. We detected the number of foci per nucleus 30 min, 3 h, 6 h, 9 h and 12 h after the acute irradiation.

Figure S4 shows the repair kinetics of radiation-induced DSBs: (A) for WI-38 cells and (B) for HBEC-3KT cells. Evaluating the statistically significant reduction of DSBs by using the Tukey-Kramer test, the induced DSBs in WI-38 cells are quickly repaired with 6 h after irradiation, whilst those in HBEC-3KT cells are slowly repaired compared with WI-38 cells. From the linear interpolation as described in Fig. S4, the half-time for repair of DSBs was 2.11 h for WI-38 cells and 3.29 h for HBEC-3KT cells, confirming that the lager number of DSBs in HBEC-3KT cells is attributed to the slower repair speed than WI-38 cells.

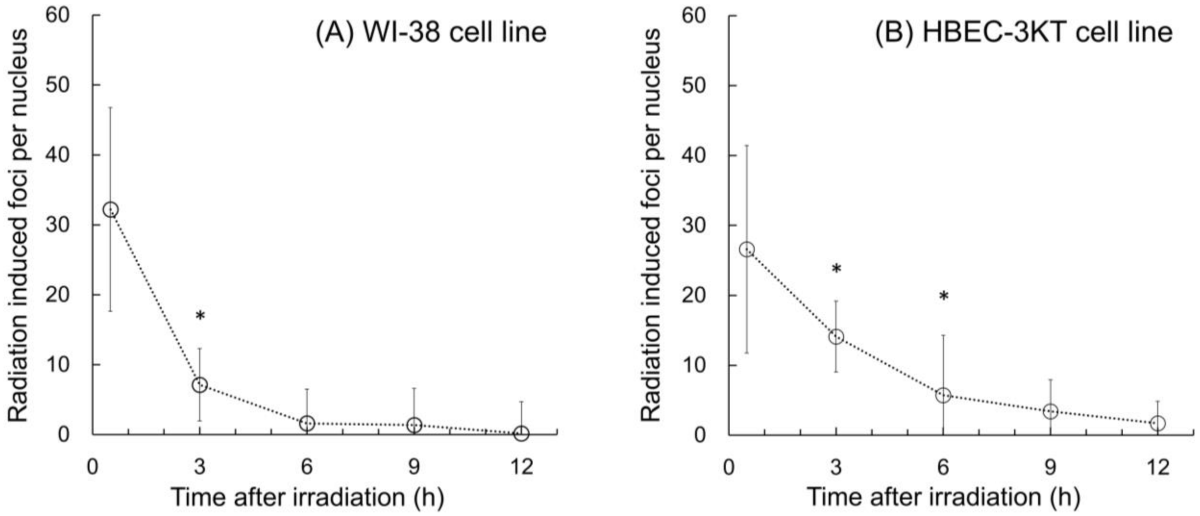

Figure S4 Kinetics for repair of DSBs: (A) WI-38 cells and (B) HBEC-3KT cells. We exposed the cells to 1.0 Gy of 6 MV-linac X-rays and detected the DSBs by means of γ-H2AX focus formation assay. The significant differences between the two groups (0.5–3 h, 3–6 h, 6–9 h and 9–12 h) were evaluated by the Tukey-Kramer method, representing the symbol* as 5 % significant differences. The half-time for repair of DSBs in WI-38 and HBEC-3KT cells were 2.11 h and 3.29 h, respectively.

Conflict of Interest

The authors declare that they have no conflict of interest.

Author Contributions

Y.M. and T.S. designed the study. Y.M. performed cell culture and *in vitro* experiments. Y.S. enclosed the Cs-bearing ball into micro-capillary. N.H. supported the experimental design and data interpretation. H.D. supported to perform *in vitro* experiments. M.I. supported the irradiation with 6 MV-linac X-rays including dose measurement. Y.M. wrote the manuscript. T.S. supervised the study. All authors reviewed the manuscript.

V. *References*

1. Matsuya, Y., Ohtsubo, Y., Tsutsumi, K. Sasaki, K., Yamazaki, R., Date, H. Quantitative estimation of DNA damage by photon irradiation based on the microdosimetric–kinetic model. *J. Radiat. Res.*, 55, 484–493 (2014).
2. Satou, Y., Sueki, K., Sasa, K., Yoshikawa, H., Nakama, S., Minowa, H., Abe, Y., Nakai, I., Ono,

T., Adachi, K., Igarashi, Y. Analysis of two forms of radioactive particles emitted during the early stages of the Fukushima Dai-ichi Nuclear Power Station accident. *Geochem. J.* 52, 137-143 (2018).

3. Sato, T., Iwamoto, Y., Hashimoto, S., Ogawa, T., Furuta, T., Abe, S., Kai, T., Tsai, P.E., Matsuda, N., Iwase, H., Shigyo, N., Sihver, L., Niita, K. Features of Particle and Heavy Ion Transport code System (PHITS) version 3.02. *J. Nucl. Sci. Technol.*1881-1248 Online (2018).
4. Endo, A., Yamaguchi, Y., Eckerman, K.F. Nuclear decay data for dosimetry calculation - Revised data of ICRP Publication 38, *JAERI* 1347 (2005).
5. Hirayama H, Namito Y, Nelson W et al. The EGS5 code system. *SLAC Report 730,* prepared for the Department of Energy, USA, 2005.
6. Lehman, A.R., Stevens, S. The production and repair of double strand breaks in cells from normal human and from patients with ataxia telangiectasia. *Biochimica et Biophysica Acta*, 474, 49-60 (1977).
7. Ager, D.D., Dewey, W.C., Gardiner, K., Harvey, W., Johnson, R.T., Waldren, C.A. The measurement of radiation-induced DNA double-strand breaks by pulsed-field gel electrophoresis. *Radiat. Res.*, 122, 181-187 (1990).
8. Hamada, N., Schettino, G., Kashino, G., Vaid, M., Suzuki, K., Kodama, S., Vojnovic, B., Folkard, M., Watanabe, M., Michael, B.D., Prise, K.M. Histone H2AX Phosphorylation in Normal Human Cells Irradiated with Focused Ultrasoft X Rays: Evidence for Chromatin Movement during Repair. *Radiats Res.* 166, 31-38 (2006).
9. Mori, R., Matsuya, Y., Yoshii, Y., Date, H. Estimation of the radiation-induced DNA double-strand breaks number by considering cell cycle and absorbed dose per cell nucleus. *J. Radiat. Res.* 59(3), 253-260 (2018).
10. Matsuya, Y., McMahon, S.J., Tsutsumi, K., Sasaki, K., Okuyama, G., Yoshii, Y., Mori, R., Oikawa, J., Kevin, M.P., Date, H. Investigation of dose-rate effects and cell-cycle distribution under protracted exposure to ionizing radiation for various dose-rates. *Sci. Rep.* 8, 8287 (2018).
11. Mao, Z., Bozzella, M., Seluanov, A., Gorbunova, V. DNA repair by nonhomologous end joining and homologous recombination during cell cycle in human cells. *Cell Cycle,* 7:18, 2902-2906 (2008).
